# 3.2 million stillbirths: epidemiology and overview of the evidence review

**DOI:** 10.1186/1471-2393-9-S1-S2

**Published:** 2009-05-07

**Authors:** Joy E Lawn, Mohammad Yawar Yakoob, Rachel A Haws, Tanya Soomro, Gary L Darmstadt, Zulfiqar A Bhutta

**Affiliations:** 1 Saving Newborn Lives/Save the Children-US, Cape Town, South Africa; 2 Center for Health and Development, Institute of Child Health, London, UK; 3 Health Systems Research Unit, Medical Research Council of South Africa; 4 Division of Maternal and Child Health, The Aga Khan University, Karachi – 74800, Pakistan; 5 Department of International Health, Bloomberg School of Public Health, Johns Hopkins University, Baltimore, Maryland, USA

## Abstract

More than 3.2 million stillbirths occur globally each year, yet stillbirths are largely invisible in global data tracking, policy dialogue and programme implementation. This mismatch of burden to action is due to a number of factors that keep stillbirths hidden, notably a lack of data and a lack of consensus on priority interventions, but also to social taboos that reduce the visibility of stillbirths and the associated family mourning. Whilst there are estimates of the numbers of stillbirths, to date there has been no systematic global analysis of the causes of stillbirths. The multiple classifications systems in use are often complex and are primarily focused on high-income countries. We review available data and propose a programmatic classification that is feasible and comparable across settings. We undertook a comprehensive global review of available information on stillbirths in order to 1) identify studies that evaluated risk factors and interventions to reduce stillbirths, 2) evaluate the level of evidence for interventions, 3) place the available evidence for interventions in a health systems context to guide programme implementation, and 4) elucidate key implementation, monitoring, and research gaps. This first paper in the series outlines issues in stillbirth data availability and quality, the global epidemiology of stillbirths, and describes the methodology and framework used for the review of interventions and strategies.

## Stillbirths – a hidden loss

Recent global estimates suggest that at least 3.2 million babies are born dead each year [1,2]. While the highest absolute numbers of stillbirths occur in South Asia, driven by the large population size of that region, the incidence rates are highest in sub-Saharan Africa. Wide variations exist: in high-income countries, stillbirth rates are below 5 per 1000 births, compared to approximately 32 per 1000 in South Asia and sub-Saharan Africa [1]. These disparities also apply within countries, since economically deprived communities have higher stillbirth rates than wealthier populations due to disparities in risk factors and inequalities in access to and quality of health care [3].

**Figure 1:**
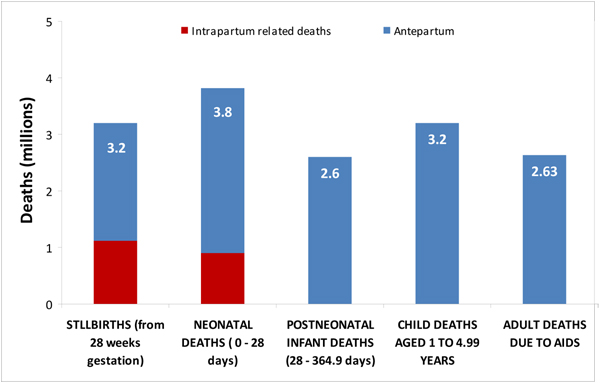
**Stillbirths – the mortality burden compared to other linked global health mortality burdens**. Data sources [1,5,6,15,55].

The overwhelming majority (98%) of stillbirths occur in low-/middle-income countries. Stillbirths are mostly uncounted in local data collection systems and are also invisible in global policy and programme priorities. This low level of attention and investment is clearly not commensurate to the large burden. In fact, the estimated numbers of stillbirths are greater than many other conditions high on the global agenda, including HIV/AIDS, for example (Figure 1). Estimates suggest that global stillbirth numbers (3.2 million) approach the total number of neonatal deaths (3.8 million) [4] and approximate the number of childhood deaths that occur after the first but before the fifth birthday (3.2 million) [1]. Intrapartum stillbirths (1 million) [5] alone exceed global child deaths due to malaria (820,000) [6] and yet attention and investment for malaria are much greater than for stillbirths.

Some health conditions lack investment because the problem only occurs in low-income countries. In contrast, for stillbirths there is increasing attention in Europe, North America and Australia with the recognition that stillbirth rate reduction has been minimal in the last decade in these countries [7]. In many high-income countries, for every neonatal death there are now approximately 1.7 stillbirths [8]. Smith and Fretts estimate that stillbirths account for 75% of all preventable perinatal deaths in these countries [7]. Relative to the size of the burden, however, stillbirths remain low on the health agenda even in high-income countries. This mismatch of burden to action is due to a number of factors that keep stillbirths hidden, notably a lack of systematic compilation of data on the numbers and cause of stillbirths, but also to social taboos affecting recognition and grieving for stillbirths. Moreover, in low-income countries and even in many high-income countries, there is a lack of consensus on the priority measures to reduce stillbirths. This lack of a well-defined programme agenda, in conjunction with lack of data and social invisibility, impede action and investment.

This paper is the first in a series about the burden of stillbirths and the evidence for efficacy of interventions to prevent stillbirths, especially in low- and middle-income countries. This first paper provides an overview of the applied epidemiology, the interventions reviewed and the search strategies and methods used. Subsequent papers analyze the evidence for interventions before and during pregnancy (papers 2 and 3) [9,10], for screening and monitoring (paper 4) [11] and during childbirth (paper 5) [12] to prevent stillbirths. The final paper reviews the evidence for interventions to prevent stillbirth in a health systems context and suggests a way forward, given the evidence assessed throughout the series, to address stillbirth prevention through policies, programmes and research [13].

### Barriers to recording and reducing stillbirths

While the world's neonatal deaths have received increasing global attention in recent years [5], stillbirths have remained virtually invisible to policymakers and funding agencies despite the fact that stillbirths have many common risk factors with neonatal deaths and maternal deaths, both of which are centrally placed in the Millennium Development Goals (MDGs). Stillbirth data are not tracked in the MDG indicators or included in the World Health Organization's (WHO's) annual World Health Report, although they will be included in the mortality tables of the next version of the Global Burden of Disease. There are many reasons for this policy invisibility, but important reasons include the lack of consistency in defining stillbirths and lack of systematic data on the rates and numbers, and especially systematic estimates for specific causes of stillbirths. Socio-cultural barriers to recognition and reporting of stillbirths play an important role, both in limited data collection but also in mobilising civil society attention. In contrast to the public outcry and street demonstrations demanding treatment that have been observed in the case of HIV, families may not even discuss intrapartum stillbirths even though these are eminently preventable.

#### Lack of clarity and consistency in definitions

Recognition of stillbirths as a public health concern is hampered by confusion and inconsistent application of definitions. The messages to policymakers and civil society regarding the size of the problem are complex and inconsistent, even for the basic definition of stillbirth. Multiple definitions are in use in different settings based on different parameters including birth weight (350, 500 or 1000 g), and/or body length or gestational age. The minimum gestational age defining a stillbirth may vary from 20 to 28 weeks of gestation. This cut-off is generally earlier in high-income countries than in low-/middle-income countries based on standards of viability. For international comparability, the WHO recommends the inclusion of all infants born dead and weighing 1000 g or more at birth (if birth weight is available), or after 28 completed weeks of gestation, or attainment of 35 cm crown-heel length. Here we will use the colloquial term, stillbirth, to refer to both early and late fetal deaths; but it should be noted that the international comparison data for stillbirth rates refer only to late fetal deaths (over 1000 g or 28 weeks gestation). We also note that the weight and gestational equivalents are approximate and some measurement bias is introduced by considering these to be equivalent.

**Figure 2:**
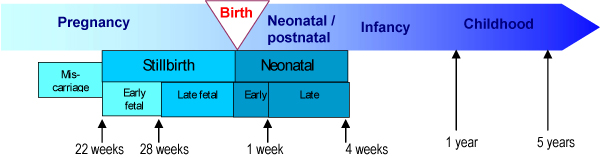
**Epidemiological time periods and definitions**. *Adapted from Lawn JE, Kerber KJ eds 2006 [56].

Additional confusion is introduced by inconsistent definitions of the portion of neonatal deaths included in the umbrella term "perinatal mortality," which has been the traditional measure used to report stillbirths and neonatal deaths. Generally this includes the stillbirth rate, which is highly variable; additionally, the neonatal component usually refers to just the first 7 days of the neonatal period (early neonatal deaths), but some definitions encompass deaths through day 28 (neonatal deaths) (Table 1 and Figure 2). Increasingly, perinatal epidemiologists are moving away from the term "perinatal mortality" and are reporting stillbirth rates and neonatal death rates separately [14]. This distinction is prerequisite for improved measurement and attention of the respective burdens of stillbirths and neonatal deaths. Whilst many data issues and programme solutions are similar, there are enough differences to justify separate tracking and both comprise a very significant mortality burden.

**Table 1:** Epidemiological definitions related to stillbirths

***Fetal death***: The International Classification of Diseases, Revision 10 (ICD-10) defines a fetal death as "*death prior to the complete expulsion or extraction from its mother of a product of conception, irrespective of the duration of pregnancy; the death is indicated by the fact that after such separation the fetus does not breathe or show any other evidence of life, such as beating of the heart, pulsation of the umbilical cord, or definite movement of voluntary muscles*" without specification of the duration of pregnancy.

***Early fetal deaths***: According to the ICD-10, an early fetal death is death to a fetus weighing at least 500 grams (or, if birth weight is unavailable, after 22 completed weeks gestation, or with a crown-heel length of 25 centimetres or more) [57].

(Birth weight is prioritised over gestational age because when ICD 10 was developed in the 1980s birth weight was believed to be more reliably reported. However globally less than half of live births are weighed and very few stillbirths are weighed, and gestational age data is more available at least based on Last Menstrual Period.)

***Late fetal deaths (stillbirths)***: A late fetal death is defined as a fetal death weighing at least 1000 grams (or a gestational age of 28 completed weeks or a crown-heel length of 35 centimetres or more) [57]. The ICD-10 recommends this definition for the purposes of international comparison.

***Stillbirths***: Stillbirth is the colloquial term commonly used term for fetal death, and is the term used in this series to refer to both early and late fetal deaths.

***Stillbirth rate***: As the data used here is for international comparison, all stillbirth rate data refer to late fetal deaths i.e. the number of babies born dead after 28 weeks of gestation per 1,000 total births.

***Early neonatal mortality rate***: The number of early neonatal deaths (deaths in the first 7 days of life) per 1,000 live births.

***Perinatal period***: This time interval includes some portion of late pregnancy and some or all of the first month of life. It has been used to refer to at least 10 different time periods depending on the time period cut offs used. The term "perinatal" is also used to refer to some, but not all causes of neonatal death in the ICD-10 [57]. Hence the term often causes confusion [14]. In this paper, we use perinatal deaths to include stillbirths after 28 weeks gestational age and early neonatal deaths in the first 7 days of life. In general, however, we have specified the outcome (stillbirth, or neonatal) or the cause of death where the data has allowed this distinction.

Adapted from ref with permission [58]

ICD refs [57,59]

Misclassification between stillbirths and early neonatal deaths poses specific measurement challenges. Some estimates suggest that 1 million stillbirths globally are intrapartum [5], and up to 50% of the world's estimated 4 million neonatal deaths occur around the time of delivery [15]. Live born infants dying in the first minutes or hours of life may be misclassified as stillbirths for a number of clinical, socio-cultural, and/or documentation reasons. Stillbirths may also be deliberately misclassified as live births (e.g., if social and maternity benefits are only given to mothers of live births). This risk of misclassification has particular importance for child survival programmes. As obstetric care improves, particularly before intensive neonatal care is instituted, historical data suggest that intrapartum stillbirth rates will fall faster than early neonatal mortality [16]. If programmes are not tracking this reduction in stillbirths, the first effect on programme data may be babies who have avoided dying as a stillbirth, but could then die as an early neonatal death, resulting in the possibility of increasing early neonatal deaths slightly but still reducing perinatal deaths overall. Without accurate data on stillbirths, tracking progress in reducing neonatal mortality will be prone to measurement and interpretation biases on the real effect of programmes.

#### Limited and poor quality data on stillbirth rates and numbers

The information gaps for stillbirths are immense. For the countries accounting for the majority of the burden, vital registration systems are unreliable or nonexistent, and indeed around two thirds occur in settings where most births are at home. Countries with vital registration data for stillbirths do not routinely report these to the United Nations. Underreporting of stillbirths in vital registration data is well documented in both high-income [17] and low-/middle-income countries. For example, in Thailand, which is considered a middle-income country, no stillbirths were reported to the vital registration system in a rural district [18]. Globally, the major sources of mortality data include intermittent large household surveys, demographic surveillance data, and clinical records. Stillbirths may be undercounted in retrospective surveys by a margin of 20 percent or more [19]. Many surveys rely on live birth histories and one simple question regarding stillbirths. There is an obvious logic for the use of pregnancy history modules in lieu of birth histories, but there is little empiric data to support increased validity or to assess the additional workload for the survey system. Obtaining these data is an urgent need, as more accurate stillbirth data collected through these large scale surveys would dramatically increase the availability of stillbirth rate data in the highest burden countries (Lawn, personal communication 2009).

Given the lack of nationally representative data for most of the world, the only stillbirth rate data for over 90% of the burden relies on national level modelling. This modelling is based on useable vital registration and survey data, as well as extensive literature searches to develop a predictive model for national stillbirth rates using national covariates as inputs [1]. WHO also derived stillbirth estimates based on multiplying national estimates of early neonatal mortality rate estimates by a factor of 1.2 since analysis of historical data from several European countries suggested this ratio of early neonatal deaths to stillbirths [2]. Both sets of estimates stress the fact that they are conservative and are likely to underestimate the true number of stillbirths. Work is in progress to produce a new set of estimates for stillbirth rates and cause of death in over 190 countries (Lawn, personal communication 2009).

#### Lack of systematic estimates for causes of stillbirths

**Figure 3:**
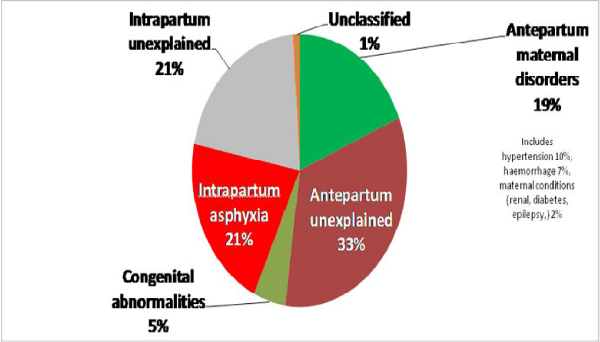
**Causes of stillbirths in Pakistan according to verbal autopsy after a nationally representative household survey**. Pakistan DHS 2006–7, Bhutta *et al. *[22].

Stillbirth cause-of-death data are available through national perinatal surveillance systems in some high-income countries. One well-known example is the United Kingdom Confidential Enquiry into Maternal and Child Health (CEMACH). The recent European Peristat report, however, highlights the lack of comparable cause-of-death data for stillbirths [8], even within Europe. South Africa is unique amongst middle-income countries in having a national Confidential Enquiry for Maternal Deaths and also a voluntary perinatal audit system which now covers over 40% of the country's births, and provides valuable data not only on direct causes of stillbirth and neonatal death, but also on delays at home and modifiable factors in the health system. [20]. To date, only two low-income countries – Egypt [21] and Pakistan [22] (Figure 3) – have reported national assessments of the causes of stillbirths in a verbal autopsy follow-up to their Demographic Health Surveys. Only recently have stillbirths been added to verbal autopsy questionnaires [23]; their value to date is limited by the lack of a comparable classification system for stillbirth cause of death, elucidated below. Overall, the cause of death data for stillbirths in low- and middle-income countries are patchy and dependent largely on special studies.

While lack of data is a large hurdle to overcome, another major barrier that could be more rapidly addressed is the lack of a classification system for low-income countries that is feasible, but maps in a comparable way onto more complex classifications. Hence two-thirds of the world's stillbirths cannot be linked to programmatically meaningful categories for prevention strategies. Stillbirth classification systems have proliferated over the years and a review suggests at least 33 are in use [24]. Most of these are designed for high-income countries and involve laboratory and pathological examination of the baby and the placenta, so are impractical for use when the only information for most stillbirths is through verbal autopsy (interview with the mother or caregiver) occurring a year or even longer after the loss.

One useful distinction for stillbirth prevention strategies is between macerated (antepartum) and fresh (intrapartum) stillbirths; importantly, this can generally be distinguished in verbal autopsy studies. Examination of fetal remains for signs of skin deterioration, skin or umbilical cord staining due to darkened amniotic fluid, or skull softening can assist in determining whether the fetus died more than 12 hours prior to delivery (macerated stillbirth) or less than 12 hours (fresh) [5]. There is some potential for misclassification between these categories. For example, in settings with major delays in access, stillbirths may die during labour, but not be delivered for days, by which time they are classified as macerated. Conversely, some intrapartum stillbirths may be due to infections or congenital causes. The extent of this misclassification may vary locally and requires more study [5]. Rates of fresh stillbirths are assumed to reflect the quality of intrapartum care (care in labour), while rates of macerated stillbirths are assumed to reflect the quality of fetal growth and of care during the antenatal period. In the published data globally, the split is 15–40% intrapartum [5], 40–60% antepartum, though this may vary in settings based on risk factors and availability and quality of intrapartum care (Lawn, personal communication 2009).

**Figure 4:**
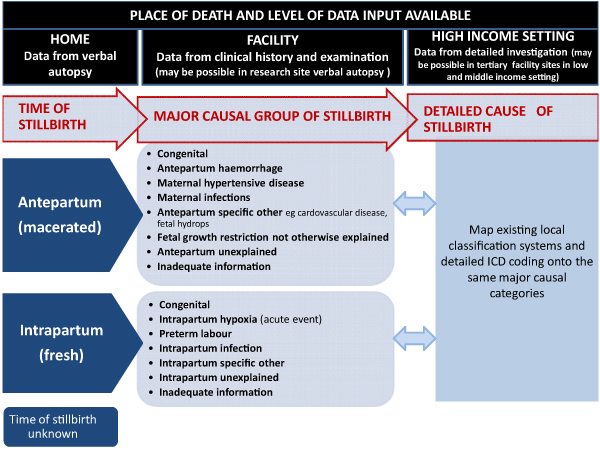
**Consistent classification for causes of stillbirths**. Source: Provisional classification system for global estimates of cause of stillbirth by the Child Health Epidemiology Reference Group (CHERG), Global Alliance for Prevention of Prematurity and Stillbirths (GAPPS) and Saving Newborn Lives/Save the Children for WHO. Some causes will be systematically missed in verbal autopsy assessments but are still important to delineate for comparability e.g. internal congenital abnormalities and maternal infections.

Once these two major time groups (antepartum and intrapartum) are defined, a more detailed set of programmatically relevant causal groups can be distinguished. This intermediate level of detail is possible with clinical data and achievable in most facility deaths in low- and middle-income countries (e.g., the South African national Saving Babies data) [25,26]. For high-income countries, many of the existing more complex classification systems that may require more investigations can be mapped onto simpler clinical categories (Figure 4). In the clinical data, some causal groups will be systematically underestimated but are still important to delineate consistently. For example, congenital abnormalities are underestimated even in high-income countries but are markedly underestimated in verbal autopsy data because only obvious external abnormalities are detected and important internal structural and metabolic disorders are missed. In the global data, around 5–15% of stillbirths are attributed to a congenital cause. Another important cause of stillbirth that is often missed is maternal syphilis.

Figure 4 proposes groupings allowing a layered approach with increasing complexity of causal attribution in varying settings according to local data capacity. This approach needs to be tested with existing data sets and refined for possible wider use, for example, in the global burden of disease estimates for stillbirth cause of death.

#### Poor understanding of mechanisms and risk factors

Risk factors and conditions associated with stillbirth overlap with those causing maternal deaths and also neonatal deaths, yet often stillbirth outcomes are not explicitly reported in studies [27]. The most important risks can be considered under the headings of those present before pregnancy, maternal medical conditions during pregnancy, exposure to harmful substances, and contextual factors affecting access to care (this last category is particularly important for intrapartum stillbirths) (Table 2). Common mechanisms include placental insufficiency, fetal damage stemming from the maternal inflammatory response, and acute fetal hypoxia. At times the distinction between risk factor and an associated condition and a direct cause becomes a continuum (Table 2). Prior stillbirth is often implicated as a risk factor for subsequent stillbirth, but it remains unclear how and in what measure environmental, physiological, socioeconomic, and genetic factors contribute to this susceptibility [28]. Since risk factors for stillbirth are also linked with negative outcomes in subsequent pregnancies, this implies that the benefits of preventing stillbirth are multiplicative. Prevention of stillbirth simultaneously benefits child survival and also reduces the chances of the mother having another high-risk pregnancy quickly after a fetal loss, which in turn increases the mother's risk.

**Table 2:** Mechanisms for stillbirth and the linked conditions and risk factors

**CONDITION OR RISK FACTOR**	**PROBABLE MECHANISMS**
**Maternal age at pregnancy or birth spacing practices**	
• Pregnancy at young age (<18 yrs)	• Increased risk of obstetric complications e.g. obstructed labour if young (<18)
• Maternal age > 35	• Increased risk of pregnancy induced hypertension in teenage pregnancies
• Short interpregnancy interval	• Increased risk of congenital anomalies, particularly chromosomal defects, with advanced maternal age
• Grand multiparity (> 4 prior pregnancies)	• Increased risk of gestational diabetes with grand multiparity

***Maternal nutritional status before pregnancy***:	
• Short maternal stature (<145 cm)	• Increased risk of feto-pelvic disproportion if malnourished in childhood
• Undernutrition (low BMI/specific Micronutrient deficiencies (eg folate)	• Increased risk of neural tube defects with folic acid deficiency
• Obesity	• Unknown pathways (e.g., obesity carries risk of gestational diabetes and pre-eclampsia, but mechanisms unknown)
• Severe anaemia	

***Maternal medical conditions during pregnancy***:	
• Diabetes	• Uncontrolled diabetes may result in macrosomia and increased risk of obstructed labour
• Hypertensive disorders (pre-eclampsia/eclampsia)	• Poorly controlled diabetes carries increased risk of congenital abnormalities
• Cholestasis or other liver disease	• Placental dysfunction including abruption (hypertension), reduced fetal growth, increased risk of acute on chronic fetal hypoxia
• Thrombophilias	• Placental abnormalities like intravascular thrombi, decidual vasculopathy and ischemic necrosis with villous infarctions (in thrombophilias)

***Exposure to harmful substances***:	
• Tobacco/alcohol/drug use	• Reduced fetal growth, increased risk of acute on chronic fetal hypoxia (increased fetal carboxyhemoglobin and vascular resistance with smoking and biomass fuels)
• Cooking fires (biomass fuel)	• Increased risk of congenital abnormalities with exposure to certain toxins or drugs, including occupational exposure such as pesticides
• Exposure to environmental toxins	

***Contextual factors: socioeconomic disadvantage and access to care, especially obstetric care***:	
• Poor access to healthcare services because of distance, and/or financial barriers	• Increased risk of obstetric complications e.g. obstructed labour if young (<18) and/or malnourished in childhood and/or FGM resulting in increased combined risk of feto-pelvic disproportion
• Ethnic or religious minority affecting equal access to care	• Increased risk of infection and undiagnosed/untreated infections
• Maternal illiteracy/low educational status	• Increased delays in accessing care
• Female genital mutilation (FGM)	• Lack of quality emergency obstetric care even when care is accessed (e.g. no caesarean section or delay to time of section, or need for additional payments)
	• Inability to afford quality obstetric care
	• Some risk factors are systematically associated with low socio-economic status (e.g., extremes of maternal age, extremes of body mass index, and smoking, alcohol and drug abuse)

Risk factors and causes for stillbirths vary between low- and high-income countries [7]. That the overwhelming majority of stillbirths occurring in low- and middle-income countries is explained in part by higher prevalence in these countries of poor obstetric care, but also a higher prevalence of risk factors, notably nutritional and birth spacing [27]. With more complex classification systems and more careful investigation, the percentage of unexplained stillbirths can be reduced. However in many studies, even in high-income countries, the cause of death may remain unknown in one-third or more of stillbirths [29].

#### Losses that are socially invisible and remain a taboo

Stillbirths are invisible at policy level partly because they are frequently invisible at the societal level (Table 3). Public announcement or acknowledgement of pregnancy loss is rare in any culture [30] although more recent media attention to such losses in the UK suggests change is possible [31]. In low-income countries, stillbirths remain a largely hidden phenomenon, as they often occur at home, fetal remains are buried without ceremony, and families rarely mourn publicly. Wherever pregnancy loss and child death are common, pregnancy and childbirth are seen as rendering women and their babies highly vulnerable to harm from disease, malevolent individuals, or spiritual forces, often until well into the postnatal period [32]. Women in many cultures conceal their pregnancies as long as possible from all but a few trusted individuals to protect themselves from harm [33], and follow elaborate dietary, sexual, and physical proscriptions during pregnancy and the postnatal period [34-37]. A stillbirth is frequently regarded as not fully human, and sometimes ritually polluted; thus, remains are disposed of secretly to minimize witnesses' vulnerability to social and supernatural harm. Women's risk of being stigmatized further suppresses women's willingness or ability to discuss a stillbirth publicly, particularly if the stillbirth may be misconstrued as induced abortion [38] or is associated with childlessness [39,40].

**Table 3:** Social norms and taboos affecting the reporting of stillbirths

***NUMERATOR – Stillbirths and early neonatal deaths are often hidden***
• Loss of "not-yet-human" babies is attributed to spiritual possession and sorcery in many traditional cultures. Hence social norms suppress grieving or even discussion for fear of the spirits causing a recurrence.
• In societies where fertility is prized, having a stillbirth may constitute failure as a wife and may result in divorce, adding a layer of shame to having had a stillbirth.
• Lack of societal recognition of a stillbirth as a loss (e.g. compared to a child death) also results in suppressed grieving and lengthened time for grief resolution.
• Women may fear being accused of having an induced abortion or not wanting the baby.
• Some cultures believe a stillbirth occurs because the woman was unfaithful during pregnancy, so the event may be concealed to prevent gossip.

***DENOMINATOR – Pregnancy is concealed in many cultures***
• Pregnant women are believed to be more vulnerable to sorcery, spirit possession, injury, and disease. Hence pregnancies are not publicly acknowledged until they "show" and may even be denied when very apparent (e.g., an Ashanti in Ghana when asked if pregnant is expected to say "No I am only drinking too much water"). In many cultures, disclosure is limited to one's partner and one or two trusted females to secure support.
• In societies with high fertility and high rates of breastfeeding, women may not be menstruating regularly and may be several months pregnant before they are aware of the pregnancy.

***DATA IMPLICATIONS***
• Underreporting of stillbirths and pregnancies is common in many settings. Sensitivity may be heightened where induced abortion is illegal or socially unacceptable.
• Mortality data collection techniques are required that are more confidential and woman-sensitive.
• An objective scoring system for stillbirth data quality is required so that falsely low rates are not used for programme priority setting and tracking of programme effectiveness.
• Analysis suggests that existing data collections systems underestimate stillbirth rates (Vital Registration systems by 34% and Demographic and Health Surveys by at least 30%). Current data in many settings may need to be adjusted using modelling techniques.

***SOCIETAL IMPLICATIONS***
• Social taboos mean that open mourning, public discussion and also media coverage is rare, and this affects the policy priority given to stillbirths by the media and by politicians.

Even where mourning is culturally suppressed, research suggests that grief responses persist. Several Western clinical psychological studies using the Perinatal Grief Scale have found that the grief and depression felt by mothers and families of a stillborn baby may exceed that associated with a neonatal death [41,42]. In many traditional societies, grieving openly is discouraged in an effort to guard against recurrent loss because grieving is thought to lead to depression, which delays physical recuperation [43]. The consequent absence of visible emotion associated with stillbirth – and sometimes neonatal deaths – in many low-income/middle-income countries has led to the premature conclusion that perinatal losses are "non-events" [39,44,45]. However, evidence from Tanzania, Cameroon, and Nepal suggests that grief responses after stillbirth are powerful even where expression is suppressed by strong social norms [46,47]. This evidence suggests a large unmet psychological need for bereaved mothers and their families. Suppressed grieving and mourning also increases the likelihood of concealment of stillbirths from researchers. These socio-cultural aspects of stillbirth are mutually reinforcing, presenting hurdles for ascertainment of stillbirths in settings where the burden is greatest.

### Stillbirths – current epidemiology to guide action

In low-income countries, stillbirth rates are between 10-fold and 20-fold higher than in middle- and high-income countries. If the coverage and quality of periconceptional, antenatal, and intrapartum (especially comprehensive obstetric) care were increased in low-income countries, stillbirth rates could be expected to decline markedly. Given that 1 million stillbirths occur during the time of labour and that half of the world's births are in facilities, improved obstetric care offers an immediate opportunity to reduce these deaths and the linked 840,000 neonatal deaths that are intrapartum-related [5]. However, many intrapartum stillbirths occur at home or on the way to a facility, so innovative approaches are required to address delays in accessing obstetric care [48,49] and to assess which interventions are feasible and scaleable to implement in the community. Around 2.2 million stillbirths occur during the last trimester, but before the onset of labour (antepartum). Given that over 75% of pregnant women globally access antenatal care (72% in Africa and 68% in South Asia [4], there are many missed opportunities for effective interventions to be provided through antenatal care. Priority conditions to address include pregnancy induced hypertension; antepartum haemorrhage; maternal infections such as syphilis, malaria and HIV; and obstetric risk conditions such as multiple pregnancy and abnormal lie. Systematic review of the wide range of interventions is required, as well as consideration of how to deliver these in the context of weaker health systems (Table 4).

**Table 4:** Stillbirths – priorities for action based on the data

**GLOBAL DATA AND POLICY PRIORITIES**
• ***Tracking mortality reduction***: Almost all (98%) of the world's 3.2 million stillbirths occur in low- or middle-income countries, yet stillbirths are rarely mentioned by global decision makers or United Nations Agencies. This is a missed opportunity for large scale maternal, newborn and child health (MNCH) investment programmes to track significant mortality benefit. Stillbirths should be included in mortality tracking wherever child and/or maternal outcomes are being assessed in household surveys or in health system or research evaluations.

• ***Intrapartum priority***: Given that 1 million stillbirths occur during the time of labour and that half of the world's births are in facilities, improved obstetric care offers an immediate opportunity to reduce these deaths and the linked 840,000 neonatal deaths that are intrapartum-related. However, many intrapartum stillbirths occur at home or on the way to a facility, so innovative approaches are required to address delays in accessing obstetric care.

• ***Effective antenatal care***: Around 2.2 million stillbirths occur during the last trimester but before the onset of labour. Given that over 75% of pregnant women globally access antenatal care (72% in Africa and 68% in South Asia), there are many missed opportunities for effective interventions to be provided through antenatal care. Priority conditions to address include pregnancy induced hypertension; antepartum haemorrhage; maternal infections such as syphilis, malaria and HIV; and obstetric risk conditions such as multiple pregnancy and abnormal lie.

**NATIONAL DATA AND PROGRAMME PRIORITIES**
• ***In many high-income countries***, stillbirth rates have not been declining at the expected rate. Improvements are possible with increased use of confidential enquiry data and attention to implement well what is known but also to innovate to address key challenges.

• ***In middle-income countries***, strengthening vital registration data for stillbirths and scaling up perinatal audit will give more data for priority setting and tracking of programme effectiveness.

• ***In low-income countries***, urgent attention should be given to how to better measure stillbirth rates in existing large-scale household surveys (for example the use of pregnancy history instead of birth history modules) and consideration of post-survey verbal autopsy to increase data on stillbirth cause of death.

• ***In all country programmes ***for maternal and neonatal health, when scaling up, specific attention should be paid to including high-impact interventions to reduce stillbirths and to tracking key indicators for quality of care such as intrapartum stillbirth rate.

• ***Research studies ***for maternal and neonatal health outcomes should consider measuring and reporting stillbirth outcomes.

## Objectives and methods for this series of papers on stillbirths

### Objectives

Systematic synthesis of evidence for interventions to prevent stillbirths in low-/middle-income countries is lacking [50]. Certain known causes, such as intrapartum hypoxia and syphilis, have reasonably well documented interventions, but lack standard intervention approaches and implementation strategies. Other causes are less well understood. Given the vacuum of information currently available, a systematic review of interventions is a crucial step in articulating a coherent approach to reducing this large burden of deaths. In order to increase global attention to stillbirths, it is important to assemble a convincing evidence base for risk factors for stillbirths and for preventive interventions, particularly in low-income countries where most stillbirths occur.

To define future research and program priorities in this area, we undertook a comprehensive global review of available information on stillbirths, and synthesised this information to:

1. Identify studies which evaluated risk factors and interventions for stillbirths

2. Evaluate the level of evidence for interventions to prevent stillbirths

3. Apply the available evidence for interventions to programmatic settings

4. Elucidate key implementation, monitoring, and research gaps.

### Methods for searches, abstraction and synthesis

**Figure 5:**
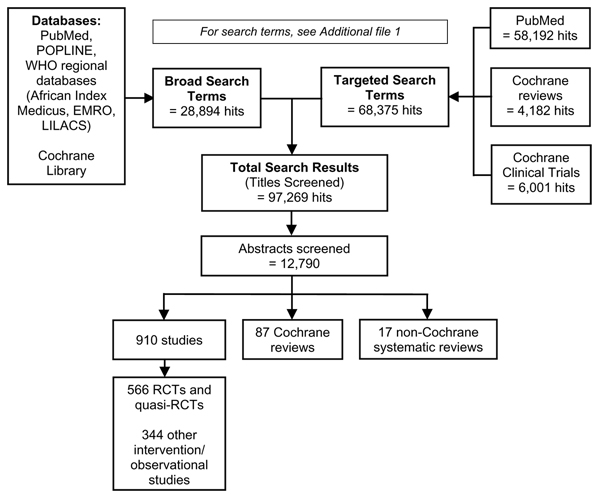
**Search strategy schematic (to March 2008)**.

We systematically evaluated all available evidence for the impact of interventions on stillbirth incidence. The search strategy is outlined in Figure 5. Searches extended to all available electronic reference libraries of indexed (PubMed/MEDLINE, POPLINE, LILACS, and WHO regional databases) and non-indexed medical journals, as well as analytical reviews and meta-analyses (Cochrane Reference Libraries). Manual reviews were conducted to incorporate relevant theses, monographs, and project documentation, including safe motherhood and child survival technical reports and evaluations. Bibliographies of available publications were scrutinised in rolling fashion to identify additional sources, including non-indexed studies and non-electronic sources.

Exhaustive search strategies were implemented using appropriate key words, accepted MeSH words, and combinations thereof. One search approach employed broad search terms (e.g., "stillbirth*", "fetal death*", or "perinatal mortality"); the other used specific search terms for interventions in combination with broad terms (e.g., amnioinfusion AND pregnancy; "fetal movement" AND "pregnancy"). Searches were restricted to references published since 1980 involving only human subjects. The detailed search terms are given in Additional file 1. Literature in languages other than English were included and reviewed by abstractors fluent in those languages. Abstracts (or full sources, if abstracts were unavailable) identified in these systematic searches were initially screened only for topical relevance and compiled in a single EndNote reference database. After duplicates were removed, the remaining body of abstracts (N = 12,790) was screened twice by two researchers according to the study eligibility criteria, detailed below.

Studies were included if they (1) detailed an intervention that could reduce stillbirth incidence through a biologically plausible pathway, and (2) reported stillbirth rate, fetal death rate, perinatal mortality rate, or data allowing calculation of such a rate as an outcome measure. For the purposes of this review, we defined stillbirth as a late fetal death after 28 weeks of gestation (Table 1 and Figure 2). We included investigator defined stillbirths in our analysis as well as intrapartum or antepartum stillbirths when so defined, but did not further disaggregate the analysis as this information was only available in a minority of reports. Several studies only reported perinatal mortality and where for specific interventions, this was the only information encompassing stillbirth outcomes, we analysed this as a surrogate outcome for stillbirths. While we did not assess interventions to avert miscarriage in this review, we included interventions that had reported impact on intrauterine deaths as an outcome some of which may include fetal loss prior to 22 weeks (Table 1 and Figure 2). Data abstracted included: stillbirth/perinatal mortality rate, number of stillbirths, and statistical significance; study variables including country, study population characteristics, date of data collection, and study design; and dependent variables.

While we prioritized randomized and quasi-randomized controlled trials, and results of analyses in the Cochrane database; the scarcity of data for many interventions, particularly from low income-country studies, prompted us to broaden our search to include less rigorous study designs, including cohort studies, case-control studies, and before-after designs. We also evaluated the data base available from a previous review of community-based interventions to address perinatal outcomes [51]. Despite this flexibility, only 1,014 reviews and studies met the review inclusion criteria.

Where new randomised controlled trials were available (after last date of search of the Cochrane reviews), we attempted meta-analyses using standard methods and software (RevMan 5, Cochrane Collaboration 2008). The inclusion and exclusion criteria were kept the same as those of the Cochrane. Where stillbirth outcomes were defined, we included these as the primary outcomes, whereas in others instances where disaggregated data were not available, perinatal deaths were analyzed. Because of paucity of information, no further analysis of the impact of interventions by income quintiles, urban/rural settings or country classification could be undertaken.

### Selection of specific interventions

The selection of interventions for the search strategy was based on biological plausibility and inclusion as a component in antenatal and intrapartum health care programmes. The various types of interventions were considered according to the time period of intervention delivery (e.g. pre-pregnancy, pregnancy, antepartum or intrapartum) and service delivery mode i.e. community, secondary level, and tertiary care health systems (Tables 5 and 6). The interventions were further analyzed according to the nature of the interventions and the continuum of the pre-pregnancy and antenatal period, and also interventions specifically related to monitoring in pregnancy and intrapartum care. We also evaluated interventions to address stillbirths through training of various cadres of health workers as well as ancillary interventions to promote their uptake.

**Table 5:** Interventions to prevent stillbirth reviewed (Papers 2 [9], 3 [10] and 4 [11])

**Care before and during pregnancy (Papers 2 **[9]** and 3 **[10])
**Family and community norms and behaviours**
Prevention of female genital mutilation and management of pregnant women with FGM
Birth spacing
Reduction of exposure to indoor air pollution
Smoking cessation
Reduction of exposure to smokeless tobacco

**Antenatal care**

**Nutritional support during pregnancy**
Periconceptional folic acid supplementation
Iron supplementation
Multiple micronutrient supplementation
Vitamin A/beta-carotene supplementation
Magnesium supplementation for deficient states
Balanced protein-energy supplementation

**Prevention and management of problems in pregnancy**
Management of hypertension in pregnancy
‧Pregnancy-induced hypertension management: calcium and anti-hypertensives
‧Anti-platelet agents in pregnancy
Heparin and other anti-coagulants
Anti-oxidants
Management of intrahepatic cholestasis
Plasma exchange
Cervical cerclage

**Infection control and treatment**
Syphilis screening and treatment
Antibiotics and anti-sepsis for high-risk pregnancies (asymptomatic bacteriuria, bacterial vaginosis and GBS colonisation)
Antibiotics for preterm rupture of membranes
Anti-helminthics
Prophylactic anti-malarials
Insecticide-treated nets
Prevention of mother-to-child transmission of HIV
Periodontal care

**Advanced monitoring and care during pregnancy (Paper 4 **[11])

**Identification and care of high-risk pregnancies**
Pregnancy risk screening
Fetal movement monitoring
Ultrasound scanning
Doppler monitoring in high-risk pregnancy
Pelvimetry
Detection and management of maternal diabetes mellitus

**Advanced monitoring in pregnancy**
Antepartum fetal heart rate monitoring with cardiotocography
Fetal biophysical test scoring
Vibroacoustic stimulation
Amniotic fluid volume assessment
Home versus hospital bed rest and monitoring for high risk pregnancies
In-hospital fetal surveillance unit

**Monitoring during the intrapartum period**
Use of the partograph
Cardiotocography with or without pulse oximetry

**Table 6:** Interventions to prevent stillbirth reviewed (Papers 5 [12] and 6 [13])

**Interventions during childbirth (intrapartum) (Paper 5 **[12])

Instrumental delivery (vacuum and forceps-assisted)
Emergency obstetric care, including Caesarean section
Induction of labour versus expectant management
Drugs for cervical ripening and induction of labour
Planned Caesarean for breech presentation
Magnesium sulphate for treatment of PIH/eclampsia or preterm labour
Maternal hyperoxygenation for suspected impaired fetal growth
Amnioinfusion

**Cross-cutting issues in the prevention of stillbirths (Paper 6 **[13])


**Community demand creation strategies**
Emergency loan and insurance funds for emergency obstetric care
Financial incentives for care seeking

**Supply side capacity building (especially human resources development)**
Training of traditional birth attendants in clean delivery and referral
Training of other cadres of community health workers
Training nurse aides (including task-shifting) as birth attendants
Training to improve skills of professional midwives in antenatal and intrapartum care
Obstetric drills
Training in neonatal resuscitation for physicians and other health care workers

**Health system organizational strategies**
Public-private partnerships to provide emergency obstetric care
Maternity waiting homes
Home birth with skilled attendance versus hospital birth for low-risk pregnancy

**Perinatal audit**

**Figure 6:**
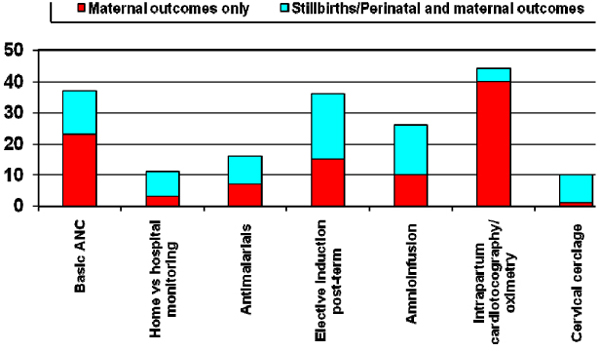
**Cochrane Library reviews of selected maternal interventions showing those that also report stillbirth outcomes**.

It must also be emphasized that our review of available evidence, especially from standard sources such as the Cochrane Library, indicated that few studies, even if plausible in terms of potential impact on birth outcomes, measured stillbirths as outcomes. Figure 6 depicts the relative proportion of RCTs in the Cochrane Library (2008) that had reported on stillbirths as outcomes indicating that for many RCTs with plausible interventions, the outcomes reported did not include stillbirths (Figure 6). Another important caveat is that most of the research took place in high-income countries. Transfer of this evidence to middle and especially low-income settings, where the cause of stillbirth and the health system capacity differ, must be undertaken with caution.

### Grading of evidence

Since the 1970s, a growing number of organizations have employed various methods to grade the quality of available scientific evidence and the strength of recommendations [51-53]. We graded the quality of each study reviewed on a scale of 1 to 4 according to the Scottish Intercollegiate Guidelines Network (SIGN) grading system [54]. All study quality ratings for each intervention were then reviewed, which allowed us to grade the cumulative assessment of the evidence for each intervention as either A, B, C or D (Table 7).

**Table 7:** Grading of Evidence Using the SIGN Grading System

**Assessment of individual studies**	**Grade**
High quality meta analysis, systematic review of randomized controlled trials (RCT), or RCT with very low risk of bias	1++
Well-conducted meta analysis, systematic review of RCTs, or RCT with a low risk of bias	1+
Meta analysis, systematic review of RCTs, or RCT with a high risk of bias	1-
High quality systematic reviews of case-control or cohort studies High quality case-control or cohort studies with a very low risk of confounding, bias, or chance and a high probability that the relationship is causal	2++
Well conducted case control or cohort studies with a low risk of confounding, bias, or chance and a moderate probability that the relationship is causal	2+
Case control or cohort studies with a high risk of confounding, bias, or chance and a significant risk that the relationship is not causal	2-
Non-analytic studies, e.g. case reports, case series	3
Expert opinion	4

**Assessment of all evidence for each intervention**	**Grade**

At least 1 meta analysis, systematic review, or RCT rated as 1++, directly applicable to the target population; or a systematic review of RCTs or a body of evidence consisting primarily of studies rated as 1+, directly applicable to the target population and demonstrating consistent overall results	A
Body of evidence including studies rated as 2++, directly applicable to the target population, and demonstrating consistent overall results; or extrapolated evidence from studies rated as 1++ or 1+	B
Body of evidence including studies rated as 2+, directly applicable to the target population and demonstrating consistent overall results; or extrapolated evidence from studies rated as 2++	C
Body of evidence 3 or 4; or extrapolated evidence from studies rated as 2+	D

Outcome measurements, including significance statistics, were evaluated for each study. Our final assessment of intervention impact considered both the magnitude and direction of reported impact, as well as the strength and number of the studies for each intervention reviewed. The recommendation as to the inclusion of specific interventions into programmes or further research was based on a Delphi process among the authors, as follows:

#### No/negative evidence of benefit

Either no statistically significant benefit of the intervention was found, or the intervention had an adverse effect with statistically significant results. Interventions in this category were not recommended for inclusion in programmes.

#### Uncertain evidence of benefit

Most or all studies reported benefit in the intervention group, but were statistically insignificant; or the results were mixed with some reporting a beneficial effect while others an adverse impact. Further research is needed before these interventions can be recommended for inclusion in programmes.

#### Some evidence of benefit

Some evidence of positive, statistically significant impact on stillbirth/perinatal outcomes was found, based on evidence in observational studies. The RCTs or meta-analyses reported insignificant benefit. Benefit in large-scale programmatic interventions, however, was largely untested for these interventions. Inclusion in maternal and perinatal health intervention programmes would be optional, but inclusion of an evaluation arm is recommended whenever interventions in this category are implemented.

#### Strong evidence of benefit

Interventions in this category had incontrovertible positive impact on stillbirths or perinatal mortality (statistically significant benefit); and, thus, were recommended for inclusion in intervention programmes for maternal and perinatal health.

### Framework for interventions and outline for the series

Tables 5 and 6 show the framework for solutions and outline of the supplement. The classification of interventions was based on potential programme relevance and implementation across the continuum of care during the pre-pregnancy period, pregnancy and childbirth. These tables show the outline of the subsequent papers in this series on stillbirths.

## Conclusion

It is clear that given the large number of deaths, equivalent or larger than many other global health priorities, stillbirths are not receiving adequate attention. There are limitations in the data, but more than enough data exist to show the size of the problem and the main priorities for focus in global policy and in national programmes. The short time that women are in labour is a time of massive risk for themselves but also for their babies and requires more investment – this time period alone results in 1 million stillbirths. The remaining 2.2 million stillbirths occur in the antenatal period, and given that over 75% of pregnant women globally attend antenatal clinics at least once, this suggests major missed opportunities to include high impact interventions (Table 4). In fact, many existing maternal newborn and child health care programmes are already providing interventions that reduce stillbirths. Not tracking stillbirths means an undervaluing of the mortality benefit of these programmes. In addition, many maternal newborn and child research studies fail to report stillbirths as an outcome – a missed opportunity to expand the evidence base.

The remainder of this series will examine the evidence for interventions for stillbirths and how these interventions could be provided through existing programmes (Tables 5 and 6). Given over 3.2 million stillbirths and the opportunity to reduce this burden at a low additional cost through existing maternal, newborn, and child health (MNCH) programmes, the low policy and programme priority given to stillbirths may be unparalleled compared to any other need in global health today. Is this a simple oversight and lack of coherent communication of the data and the solutions? Or do stillbirths not count?

## List of abbreviations used

AIDS: Acquired immune deficiency syndrome; CEMACH: Confidential Enquiry into Maternal and Child Health (UK); CHERG: Child Health Epidemiology Reference Group; CTG: cardiotocography; DHS: Demographic Health Survey; GAPPS: Global Alliance for Prevention of Prematurity and Stillbirths; HIV: human immunodeficiency virus; ICD-10: International Classification of Diseases – Revision 10; MDGs: Millennium Development Goals; MNCH: maternal, newborn, and child health; RCT: randomized controlled trial; SB: stillbirth; SIGN: Scottish Intercollegiate Guidelines Network; WHO: World Health Organization

## Competing interests

The authors declare that they have no competing interests.

## Authors' contributions

The paper was written and reviewed by all the authors.

## Supplementary Material

Additional file 1 **Web Table 1: Search terms used (Completed March 2008)**. Contains terms used in the literature search for this review. Click here for file
