# Supplementary material for: 3.2 million stillbirths: epidemiology and overview of the evidence review
Source: BMC Pregnancy Childbirth. 2009 May 7;9(Suppl 1):S2. doi: 10.1186/1471-2393-9-S1-S2 (PMC2679408; doi:10.1186/1471-2393-9-S1-S2)
Supplement: Additional file 1 — Web Table 1: Search terms used (Completed March 2008). Contains terms used in the literature search for this review. [file 1471-2393-9-S1-S2-S1.doc]

***Broad Terms (PubMed, Cochrane, Popline, WHO regional databases)***

“Stillbirth*”

“Fetal death*”

“Perinatal mortality”

“Perinatal death*”

***Targeted Terms (PubMed and Cochrane Library)***

“Female genital mutilation”

“Female circumcision”

“Birth spacing” AND (stillbirth* OR “perinatal mortality”)

“Indoor air pollution” AND “pregnancy”

“Smoking cessation” AND “pregnancy”

(Maternal OR pregnancy) AND (tobacco OR nicotine) AND (stillbirth* OR “perinatal mortality” OR “fetal loss” or abortion* OR miscarriage* OR “fetal mortality” OR “fetal death*”)

“Antenatal care”

“Folic acid” AND “supplementation” AND “pregnancy”

“Iron” AND “supplementation” AND “pregnancy”

“Multiple micronutrient” AND “supplementation” AND “pregnancy”

“Multivitamin” AND “supplementation” AND “pregnancy”

“Vitamin A” AND “supplementation” AND “pregnancy”

“Magnesium” AND “supplementation” AND “pregnancy”

“Balanced energy” AND “supplementation” AND “pregnancy”

“Balanced protein” AND “supplementation” AND “pregnancy”

“Antihypertensive*” AND “pregnancy”

“Calcium” AND “supplementation” AND “pregnancy”

“Aspirin” AND “pregnancy”

“Heparin” AND “pregnancy”

“Antioxidant*” AND “pregnancy”

“Intrahepatic cholestasis” AND “pregnancy”

“Plasma exchange” AND “pregnancy”

“Cervical cerclage” AND “pregnancy”

“Syphilis” AND “screening” AND “pregnancy”

“Syphilis” AND “treatment” AND “pregnancy”

“Antibiotic*” AND “pregnancy”

“Antihelminthic*” AND “pregnancy”

“Mebendazole” AND “pregnancy”

“Antimalarial*” AND “pregnancy”

“Insecticide” AND “nets” AND “pregnancy”

“Antiretroviral*” AND “pregnancy”

“Periodontal” AND “pregnancy”

“Risk screening” AND “pregnancy”

“Fetal movement” AND “pregnancy”

“Ultrasound” AND “pregnancy” AND “pregnancy outcome”

“Ultrasound” AND “pregnancy” AND “stillbirth*”

“Ultrasound” AND “pregnancy” AND “fetal death*”

“Ultrasound” AND “pregnancy” AND “perinatal mortality”

“Ultrasound” AND “pregnancy” AND “perinatal death*”

“Doppler” AND “pregnancy”

“Pelvimetry” AND “pregnancy”

“Management” AND “gestational diabetes”

“Fetal biophysical” AND “pregnancy”

“Cardiotocography” AND “pregnancy”

“Vibroacoustic” AND “pregnancy”

“Amniotic fluid volume” AND “pregnancy”

“Home” AND “hospital” AND “monitoring” AND “pregnancy”

“Fetal surveillance unit” AND “pregnancy”

“Partogram” AND “pregnancy”

“Partograph” AND “pregnancy”

“Pulse oximetry” AND “pregnancy”

“Instrumental deliveries”

“Emergency obstetric care”

“Caesarean” AND “breech”

“Induction” AND “labour”

“Oxygen” AND “pregnancy”

“Amnioinfusion” AND “pregnancy”

“Magnesium sulphate” AND “pregnancy”

“Emergency loan funds”

“Traditional birth attendant*” AND “pregnancy”

“Community health worker*” AND “pregnancy”

“Nurse aides” AND “pregnancy”

“Midwives” AND “pregnancy outcome”

“Midwives” AND “stillbirth*”

“Midwives” AND “fetal death*”

“Midwives” AND “perinatal mortality”

“Midwives” AND “perinatal death*”

("emergencies" OR "emergency") AND ("obstetric") AND (drill OR drills OR simulation OR simulations OR simulate*)

“Health professionals” AND “pregnancy”

“Maternity waiting home*”

“Home” AND “hospital” AND “birth*”

“Perinatal audit*”
